# Oxidative stress and plasma lipoproteins in cancer patients

**DOI:** 10.1590/S1679-45082014RC3110

**Published:** 2014

**Authors:** Fernanda Maria Machado Maia, Emanuelly Barbosa Santos, Germana Elias Reis

**Affiliations:** 1 Universidade Estadual do Ceará, Fortaleza, CE, Brazil.

**Keywords:** Chronic disease, Neoplasms, Lipoproteins, Lipids, Oxidative stress

## Abstract

**Objective:**

To evaluate the relation between oxidative stress and lipid profile in patients with different types of cancer.

**Methods:**

This was an observational cross-sectional. A total of 58 subjects were evaluated, 33 males, divided into two groups of 29 patients each: Group 1, patients with cancer of the digestive tract and accessory organs; Group 2 patients with other types of cancers, all admitted to a public hospital. The plasma levels (lipoproteins and total cholesterol, HDL, and triglycerides, for example) were analyzed by enzymatic kits, and oxidative stress based on thiobarbituric acid-reactive substances, by assessing the formation of malondialdehyde.

**Results:**

In general the levels of malondialdehyde of patients were high (5.00μM) as compared to 3.31μM for healthy individuals. The median values of lipids exhibited normal triacylglycerol (138.78±89.88mg/dL), desirable total cholesterol values (163.04±172.38mg/dL), borderline high LDL (151.30±178.25mg/dL) and low HDL (31.70±22.74mg/dL). Median HDL levels in Group 1 were lower (31.32mg/dL) than the cancer patients in Group 2 (43.67mg/dL) (p=0.038). Group 1 also showed higher levels of oxidative stress (p=0.027).

**Conclusion:**

The lipid profile of patients with cancer was not favorable, which seems to have contributed to higher lipid peroxidation rate, generating a significant oxidative stress.

## INTRODUCTION

Cancer is the name given to a set of over one hundred diseases with a common feature: disorderly growth of cells that invade tissues and organs. It is regarded as a public health problem in Brazil. National estimates for 2014 point to 76 thousand new cases of cancer, including non-melanoma skin cancer.^(1)^ There are many causes, which could be external (environment and habits) or pertaining to the inner body (genetically determined), and interrelated.^(2)^

Among the mechanisms related to the pathogenesis of non-communicable chronic diseases, including cancer, we have changes caused by oxidative metabolism.^(3)^ The generation of free radicals is a continuous and physiological cell process.^(4)^ In adequate proportions, its production will enable the generation of energy through adenosine triphosphate (ATP), phagocytosis, cell growth regulation and the participation in defense mechanisms during the infectious process. However, their excessive production shows harmful effects.^(5)^

Nonetheless, an imbalance between reactive species concentrations and antioxidant defense mechanisms in the body, favoring the first, will result in oxidative stress. The harmful effects of this process are membrane lipid peroxidation and damage caused to tissue and membrane proteins, to enzymes, to carbohydrates, in addition to oxidative damage to deoxyribonucleic acid (DNA).^(6,7)^

The metabolism of cancer patients changes gradually, affecting all metabolic pathways. As for carbohydrate metabolism, tumors show an excessive glucose consumption, causing glucose intolerance, with peripheral insulin resistance.^(8,9)^ Protein catabolism is present, while most of the times there is a massive loss of musculoskeletal tissue in those patients. This event is related to a sulfated glycoprotein called proteolysis-inducing factor (PIF). This entire scenario contributes to the cachectic state very often encountered in cancer patients.^(10)^ As for lipid metabolism, inhibition of plasma lipoprotein lipase activity leads to hyperlipidemia.^(11)^ Increase of lipolysis associated with lowered lipogenesis; and increased turnover of glycerol, free fatty acids, and triacylglycerols (which are depleted from the adipose tissue) are all metabolic changes induced by advanced tumors, and they may be related to an increase of hormone-sensitive lipase (HSL) and to the release of lipolytic tumor factors.^(11,12)^ Lipolytic activity can also be mediated by the lipid mobilization factor (LMF).^(10)^

## OBJECTIVE

To assess the relation between oxidative stress and lipid profile in patients with different types of cancer.

## METHODS

This was an observational, cross-sectional study involving cancer patients admitted to the *Hospital Geral Dr. César Cals*, in the city of Fortaleza (CE). This study has been reviewed and approved by the Ethics Committee of the abovementioned hospital (protocol 497/2011 and CAAE 0011.0.041.038-11). All research subjects participated voluntarily and signed the Informed Consent. The study was conducted from July 2011 to July 2012.

The inclusion criteria were patients with all types of cancer and aged over 18 years. The exclusion criterion was having other non-communicable chronic diseases. The convenience sample consisted of 58 patients.

After patients gave their consent to join the research, a 1mL sample of plasma was requested from the clinical laboratory at the abovementioned hospital in order to determine oxidative stress and plasma lipoprotein levels.

Lipid peroxidation (oxidative stress) was determined based on thiobarbituric acid reactive substances (TBARS),^(13)^ with changes. The results were calculated according to a standard curve made from malondialdehyde (MDA) at 4µM. As an MDA reference value we considered that of healthy individuals, which is 3.31µM.^(14)^

Total cholesterol, triacylglycerol and high-density lipoprotein cholesterol (HDL-c) were determined through enzymatic methods, using commercial Labteste^®^ reagents, while all tests were performed twice.

Total cholesterol, triacylglycerol and HDL-c levels were measured using 10:10:50µL, respectively, from the plasma sample, with 1mL of the respective work reagent for each lipoprotein. After 10 minutes in a heated bath at 37°C, we measured the sample absorbance and the absorbance of each standard at 500-510nm, using a digital Photonics^®^ spectrophotometer. For HDL-c dosage, we first homogenized 250µL of plasma and 250µL of the reagent. They were centrifuged at 3,500 x g for 15 minutes and then the clear supernatant, with HDL-c, was pipetted for the assay. Low-density lipoprotein cholesterol (LDL-c) levels were obtained through the equation:^(15)^

LDL-c = total cholesteral – HDL-c – triacylglycerols/5, valid for triacylglycerols <400mg/dL

The results were classified following the National Cholesterol Education Program (NCEP) values, according to which total cholesterol levels <200mg/dL are desirable; levels ranging from 200 to 239mg/dL are borderline high; and levels ≥240mg/dL are high. For triacylglycerols, values are considered normal when <150mg/dL; borderline when ranging from 150 to 199mg/dL; high when ranging from 200 to 499mg/dL; and very high when ≥500mg/dL. For HDL cholesterol, levels were considered low when <40mg/dL; normal when ranging from 40 to 59mg/dL; and high if ≥60mg/dL. For LDL cholesterol, values described as optimal were <100mg/dL; borderline optimal when ranging from 100 to 129mg/dL; borderline high when ranging from 130 and 159mg/dL; high when ranging from 160 to 189mg/dL; and very high if >190mg/dL.^(16)^

Statistical analyses were performed using the software Statistical Package for Social Sciences (SPSS^®^), version 21. Through the Kolmogorov-Smirnov test we observed that continuous data had normal distribution. In order to analyze median differences in oxidative stress, total cholesterol, HDL-c, LDL-c and triacylglycerols, all 58 subjects in the sample were divided into two groups of 29 patients: Group 1, with cancer in the gastrointestinal tract and accessory organs, including stomach (13), pancreas (5), esophagus (3), colon (3), cecum (1), rectum (1), pharynx (1), intestine (1) bile ducts (1); Group 2, patients with other types of cancer, including hematologic (11), female genital organs (5), prostate (3), bladder (2), lung (2), thyroid (2), mediastine (2), placenta (1) and kidney (1) cancer. Medians were compared by the Student’s *t* test. Discrete variables obtained through counting were analyzed according to frequency dispersion by the χ^2 ^test. When the amount of data available was lower than the minimum limit for χ^2^, we applied the Fisher’s exact test. Results were expressed as median±standard deviation, while p<0.05 was defined as statistically significant.

## RESULTS

Among the patients we studied, there was a higher prevalence of stomach (22.4%), hematologic (19.0%), pancreatic and female genital organ (with 8.6% each) cancer, and of esophageal, colon and prostate cancer (with 5.1% each).

We also noticed a higher prevalence of gastric cancer in that sample, with predominance in the male sex (56.9%).

The oxidative stress median was 5.00μM, with 63.8% of individuals showing high oxidative stress levels. As for patients’ lipid profile, we observed the following factors: triacylglycerol median was normal (138.78±89.88mg/dL), total cholesterol was at desirable levels (163.04±172.38mg/dL), LDL-c was borderline high (151.30±178.25mg/dL) and HDL-c was low (31.70±22.74mg/dL) (Table 1).

However, 27.6% (16/58) of cancer patients exhibited total cholesterol values classified as borderline high or high; 58.6% (34/58) exhibited low HDL-c levels; and 29.3% (17/58) showed high triacylglycerol levels (Table 1).

**Table 1:** Distribution of cancer patients seen at *Hospital Geral Dr. César Cals*, from July 2011 to July 2012, according to the variables studied

Variables	Median (n=58)	Group 1 n (%)	Group 2 n (%)	p value*
Malondialdehyde (μM)	5.00±4.07			
Normal	21 (36.2%)	9 (42.9)	12 (57.1)	0.412
High	37 (63.8%)	20 (54.1)	17 (45.9)	
Total cholesterol (mg/dL)	163.04±172.32			
Desirable	42 (72.4%)	21 (50.0)	21 (50.0)	0.865
Borderline high	5 (8.6%)	3 (60)	2 (40)	
High	11 (19.0%)	5 (45.5)	6 (54.5)	
HDL-c (mg/dL)	31.70±22.74			
Low	34 (58.6%)	22 (64.7)	12 (35.3)	0.019
Normal	14 (24.2%)	3 (21.4)	11 (78.6)	
High	10 (17.2%)	4 (40.0)	6 (60.0)	
Triacylglycerol (mg/dL)	138.78±89.88			
Normal	31 (53.5%)	17 (54.8)	14 (45.2)	0.688
Borderline	10 (17.2%)	4 (40.0)	6 (60.0)	
High	17 (29.3%)	8 (47.1)	9 (52.9)	
LDL-c (mg/dL)	151.30±178.25			
Optimal	12 (20.7%)	6 (50.0)	6 (50.0)	0.296
Borderline optimal	11 (19.0%)	3 (27.3)	8 (72.7)	
Borderline high to very high	35 (60.3%)	20 (57.1)	15 (42.9)	

* χ^2 ^Test. Group 1 formed by individuals with gastrointestinal cancer; Group 2 formed by individuals with other types of cancer. HDL-c: high-density lipoprotein cholesterol; LDL-c: low-density lipoprotein cholesterol.

When comparing Group 1 (n=29), which had gastrointestinal and accessory organ cancer, with Group 2 (n=29), which included patients with other types of cancer, an association was observed between the higher prevalence of individuals in Group 1 with low HDL-c than in Group 2 (p=0.019) (Table 1).

With respect to differences in median HDL-c values, Group 1 had lower levels (31.32mg/dL) than cancer patients in Group 2 (43.67mg/dL) (p=0.038). Higher oxidative stress values were observed in Group 1 patients, with 6.83µM, while Group 2 had 4.46µM (p=0.027) (Table 2).

**Table 2:** Median malondialdehyde and lipoprotein values of cancer patients seen at *Hospital Geral Dr. César Cals*, from July 2011 to July 2012

Biochemical parameters	Group 1	Group 2	p value*
Malondialdehyde (μM)	6.83±4.92	4.46±2.57	0.027
Total cholesterol (mg/dL)	209.36±161.73	210.82±185.30	0.975
Triacylglycerol (mg/dL)	141.85±86.36	146.48±94.76	0.878
HDL-c (mg/dL)	31.32±21.18	43.67±22.92	0.038
LDL-c (mg/dL)	206.40±171.24	196.45±187.90	0.834

* Student’s *t* test. HDL-c: high-density lipoprotein cholesterol; LDL-c: low-density lipoprotein cholesterol.

## DISCUSSION

This study had important limitations with respect to its population and sample. The defined population was limited to a single study location, and we did not choose a specific type of cancer, which could yield more accurate and applicable results for the general population. Another important limitation is the sample size, since we did not calculate a representative sample and cannot make generalizations for individuals in general terms.

This higher prevalence of gastrointestinal cancer and high occurrence of female genital organ cancer in our sample are probably due to the fact that ours is a tertiary, high-complexity reference hospital in the fields of surgery, internal medicine, gynecology, obstetrics and neonatology.^(17) ^Gastric cancer affects more men, at a 2:1 ratio, and the onset is usually after 40 years old, with its incidence peaking from 50 to 70 years old. It also affects lower socioeconomic brackets and those with lower schooling levels.^(18)^

In a review article about nutritional therapy in gastric cancer, researchers observed that there are two risk factors for this type of neoplasm: constitutional, or intrinsic, and environmental, or extrinsic risk. Diet is listed as the most relevant environmental risk,^(19)^ and alcohol is also mentioned as a risk factor, as well as a state of hypochloridia or achlorhydria caused by medication, which causes pH levels that are more favorable to the formation of bacterial colonies, making *Helicobacter pylori *infections much easier. This promotes a chronic inflammatory process, which culminates in the onset of cancer. Oxidative stress induced by inflammation operates as a mediator for the formation or activation of cancer particles, causing DNA damage and playing an important role in that carcinogenesis process.^(20)^

The results obtained from oxidative stress were high, with a median value of 5.00 μM when compared with 3.31μM of healthy adult individuals.^(14)^ When antioxidant control mechanisms are exhausted or saturated, cell redox potential moves toward oxidative stress. This, in turn, will increase the potential for damage to nucleic acids, lipids or proteins, and when that DNA damage is not repaired, it can result in mutations, establishing the role of oxidative nuclear DNA damage in cancer.^(21)^

As for the lipid profile of the population studied, the low HDL-c median agrees with the literature. In a systematic review, researchers observed an inverse significant association between HDL-c and the incidence of risk of cancer. This association does not depend on LDL-c, age, body mass index (BMI), diabetes and smoking.^(22) ^Low HDL-c levels may be associated with the epidemiology of risk of developing lung cancer and with the progression of local tumors to metastatic ones.^(23)^

HDL-s is known for its antiatherogenic role in the body and, together with C-reactive protein, shows promising characteristics as a predictor of cardiovascular mortality.^(24)^

The antiatherogenic role of HDL is linked to reverse cholesterol transport (RCT), including antioxidant activities, both *in vitro *and *in vivo,* and anti-inflammatory and anti-thrombotic activities.^(25,26) ^This activity is also related to the presence of several apolipoproteins (Apo AI, apoE, apoJ, apoA-II and apo A-IV) and the following enzymes with antioxidant properties: serum paraoxonase (PON1), platelet-activating factor acetylhydrolase (PAF-AH), lecithin-cholesterol acyltransferase (LCAT) and glutathione peroxidase (GSPx).^(27)^ However, HDL can change in certain diseases and such modifications may alter its functions.^(28)^

Still comparing Groups 1 and 2, we observed greater oxidative stress in Group 1, which may be associated with poor prognosis for pancreatic and esophageal cancer, related to the invasive aggressiveness of those tumors. Despite its low incidence, pancreatic cancer has high mortality, even with an early diagnosis, just as esophageal cancer, whose 5-year survival is <10%.^(29)^

## CONCLUSION

Most patients in the sample were male and exhibited higher oxidative stress than healthy individuals, desirable total cholesterol levels, borderline high LDL-cholesterol and low HDL-cholesterol. The latter lipoprotein was also classified as even lower among patients with gastrointestinal and accessory organ cancer. Those patients also had higher levels of oxidative stress. Therefore, their lipid profile was unfavorable, especially with respect to LDL-cholesterol and HDL-cholesterol, which seems to have contributed to a higher lipid peroxidation rate. Further studies are needed to clarify possible relations between oxidative stress and lipid profiles in gastrointestinal cancer.
