# Supplementary material for: Oxidative stress and plasma lipoproteins in cancer patients
Source: Einstein (Sao Paulo). 2014 Oct-Dec;12(4):480–4. doi: 10.1590/S1679-45082014RC3110 (PMC4879916; doi:10.1590/S1679-45082014RC3110)
Supplement: Translation

# Translation: Estresse oxidativo e lipoproteínas plasmáticas em pacientes com câncer

**Authors:** Fernanda Maria Machado Maia, Emanuelly Barbosa Santos, Germana Elias Reis

**Affiliations:** 1 Universidade Estadual do Ceará, Fortaleza, CE, Brasil.

**Keywords:** Doença crônica, Neoplasias, Lipoproteínas, Lipídeos, Estresse oxidativo

## Abstract

**Objetivo:**

Avaliar a relação entre estresse oxidativo e perfil lipídico em pacientes com diferentes tipos de câncer.

**Métodos:**

Estudo do tipo observacional transversal. Foram avaliados 58 indivíduos, sendo 33 do sexo masculino, divididos em dois grupos de 29 pacientes cada: o Grupo 1 foi formado por pacientes com câncer do trato digestivo e órgãos anexos; o Grupo 2 foi formado por pacientes com outros tipos de câncer, todos internados em um hospital público. Foram analisadas concentrações plasmáticas (colesterol total, HDL e triacilglicerol, entre outras), por meio de *kits* enzimáticos, e o estresse oxidativo, com base em substâncias reativas ao ácido tiobarbitúrico, por meio da formação de malondialdeído.

**Resultados:**

Na mediana, os pacientes apresentaram valores altos de malondialdeído (5,00μM) quando comparados ao valor de 3,31μM para indivíduos saudáveis. A mediana do perfil lipídico exibiu valores de triacilgliceróis normais (138,78±89,88mg/dL), colesterol total com valores desejáveis (163,04±172,38mg/dL), LDL com limiar elevado (151,30±178,25mg/dL) e HDL baixo (31,70±22,74mg/dL). Os níveis medianos de HDL do Grupo 1 foram mais baixos (31,32mg/dL) do que dos pacientes oncológicos do Grupo 2 (43,67mg/dL) (p=0,038). O Grupo 1 apresentou níveis mais altos de estresse oxidativo (p=0,027).

**Conclusão:**

O perfil lipídico de pacientes com câncer apresentou-se desfavorável, o que parece ter contribuído para uma maior taxa de peroxidação lipídica, gerando um significativo estresse oxidativo.

## INTRODUÇÃO

Câncer é o nome dado a um conjunto de mais de cem doenças que têm em comum o crescimento desordenado de células que invadem tecidos e órgãos, sendo considerado um problema de saúde pública no Brasil. Estimativas nacionais apontam, para 2014, 76 mil novos casos da doença, incluindo os casos de pele não melanoma.^(1)^ As causas são variadas, podendo ser externas (meio ambiente e hábitos) ou internas ao organismo (determinadas geneticamente), e inter-relacionadas.^(2)^

Dentre os mecanismos relacionados à patogênese das doenças crônicas não transmissíveis, incluindo o câncer, estão as alterações causadas pelo metabolismo oxidativo.^(3)^ A geração de radicais livres é um processo celular contínuo e fisiológico.^(4)^ Sua produção, em proporções adequadas, possibilita a geração de energia pela adenosina trifosfato (ATP), fagocitose, regulação do crescimento celular e participação nos mecanismos de defesa durante o processo de infecção. No entanto, sua produção excessiva apresenta efeitos prejudiciais.^(5)^

Não obstante, o desequilíbrio entre as concentrações de espécies reativas e os mecanismos de defesa antioxidante no organismo, em favor das primeiras, resulta em estresse oxidativo. Os efeitos prejudiciais desse processo são a peroxidação dos lipídios de membrana, e a agressão às proteínas dos tecidos e das membranas, às enzimas, aos carboidratos, além de dano oxidativo ao ácido desoxirribonucleico (DNA).^(6,7)^

O metabolismo do paciente com câncer se altera gradativamente, afetando todas as vias metabólicas. Quanto ao metabolismo dos carboidratos, ocorre um consumo excessivo de glicose pelo tumor, causando intolerância à glicose, com resistência periférica à ação da insulina.^(8,9)^ O catabolismo proteico se faz presente, havendo, na maioria das vezes, uma perda maciça do tecido musculoesquelético nesses pacientes. Tal evento está relacionado a uma glicoproteína sulfatada intitulada fator indutor de proteólise (PIF, sigla do inglês *proteolysis-inducing factor*). Todo esse quadro contribui com o estado de caquexia, presente na maioria das vezes nos pacientes com patologia oncológica.^(10)^ Já no metabolismo dos lipídios, a inibição da atividade da enzima lipase lipoprotéica plasmática leva à hiperlipidemia.^(11)^ Aumento da lipólise associada à redução da lipogênese; e aumento do *turnover *de glicerol, de ácidos graxos livres, e de triacilgliceróis (que são depletados do tecido adiposo) são alterações metabólicas induzidas pelos tumores avançados, que podem estar relacionadas ao aumento da lipase hormônio-sensível (HSL, sigla do inglês *hormone-sensitive lipase*) e à liberação de fatores tumorais lipolíticos.^(11,12)^ A atividade lipolítica também pode ocorrer mediada pelo fator de mobilização de lipídios (FML).^(10)^

## OBJETIVO

Avaliar a relação entre estresse oxidativo e perfil lipídico em pacientes com diferentes tipos de câncer.

## MÉTODOS

Tratou-se de um estudo do tipo observacional transversal, envolvendo pacientes com câncer, internados no Hospital Geral Dr. César Cals, da cidade de Fortaleza (CE). O estudo foi analisado e aprovado pelo Comitê de Ética do referido hospital (protocolo 497/2011 e CAAE 0011.0.041.038-11). Todos os sujeitos da pesquisa, voluntários, assinaram o Termo de Consentimento Livre e Esclarecido. O trabalho foi desenvolvido do período de julho de 2011 a julho de 2012.

Os critérios de inclusão foram: pacientes com todos os tipos de câncer e com idade superior a 18 anos. Portar outras doenças crônicas não transmissíveis constituiu critério de exclusão. A amostra de conveniência foi formada por 58 pacientes.

Após o consentimento do paciente de sua participação na pesquisa, uma alíquota de 1mL de plasma era solicitada ao laboratório de análises clínicas do referido hospital para determinação do estresse oxidativo e das lipoproteínas plasmáticas.

A determinação da peroxidação lipídica (estresse oxidativo) foi baseada nas espécies reativas ao ácido tiobarbitúrico (TBARS),^(13)^ com modificações. Os resultados foram calculados de acordo com uma curva padrão feita com malondialdeído (MDA) a 4µM. Como valor de referência de MDA, foi considerado de indivíduos saudáveis, que é de 3,31µM.^(14)^

As concentrações de colesterol total, triacilglicerol e lipoproteína de alta densidade colesterol (HDL-c) foram determinadas por meio de métodos enzimáticos, utilizando-se reagentes comerciais da marca Labteste^®^, sendo que todas as dosagens foram realizadas em duplicata.

As dosagens de colesterol total, triacilglicerol e HDL-c foram realizadas utilizando-se 10:10:50µL, respectivamente, da amostra do plasma, com 1mL do reagente de trabalho respectivo para cada lipoproteína. Depois de 10 minutos em banho-maria a 37°C, foi realizada a leitura da absorbância da amostra e de cada padrão a 500-510nm, em espectrofotômetro digital, da marca Photonics^®^. Para a dosagem do HDL-c, 250µL do plasma e 250µL do reagente precipitante foram primeiramente homogeneizados, centrifugados a 3.500 x g durante 15 minutos e, então, o sobrenadante límpido, contendo o HDL-c, foi pipetado para o ensaio. A concentração da lipoproteína de baixa densidade colesterol (LDL-c) foi obtida por meio da equação:^(15)^

LDL-c = colesterol total – HDL-c – triacilglicerois/5, sendo válida para triacilglicerois <400mg/dL

Os resultados foram classificados de acordo com os valores do *National Cholesterol Education Program *(NCEP), que considera desejáveis valores de colesterol total <200mg/dL; limítrofes altos de 200 a 239mg/dL; e altos ≥240mg/dL. Para triacilglicerois, os valores são considerados normais se <150mg/dL; limítrofes se de 150 a 199mg/dL; altos de 200a 499mg/dL; e muito altos se ≥500mg/dL. Para HDL colesterol, os valores foram considerados baixos se <40mg/dL; normais de 40 a 59mg/dL; e altos se ≥60mg/dL. Para LDL colesterol, os valores descritos como ótimos foram <100mg/dL; limiares ótimos entre 100 e 129mg/dL; limiares elevados entre 130 e 159mg/dL; elevados entre 160 e 189mg/dL; e muito elevados se >190 mg/dL.^(16)^

As análises estatísticas foram realizadas utilizando o programa *Statistical Package for Social Sciences* (SPSS^®^), versão 21. Por meio do teste de Kolmogorov-Smirnov, foi observado que os dados contínuos apresentaram distribuição normal. Para análise das diferenças das medianas de estresse oxidativo, colesterol total, HDL-c, LDL-c e triacilglicerois, os 58 participantes da amostra foram divididos em dois grupos de 29 pacientes: Grupo 1, com câncer do trato digestivo e órgãos anexos, incluindo câncer de estômago (13), pâncreas (5), esôfago (3), cólon (3), ceco (1), reto (1), faringe (1), intestino (1) e de vias biliares (1); Grupo 2, pacientes com outros tipos de câncer, incluindo hematológicos (11), órgãos genitais femininos (5), próstata (3), bexiga (2), pulmão (2), tireoide (2), mediastino (2), placenta (1) e rim (1). As medianas foram comparadas pelo teste *t* de Student. As variáveis discretas, obtidas por contagem, foram analisadas por dispersão de frequência pelo teste χ^2^. Quando o número de informações disponíveis era inferior ao limite mínimo para o χ^2^, foi aplicado o teste exato de Fisher. Os resultados foram expressos como mediana±desvio padrão, sendo o valor de p<0,05 definido como estatisticamente significante.

## RESULTADOS

Dentre os pacientes estudados, observou-se maior prevalência de câncer do estômago (22,4%), hematológico (19,0%), de pâncreas e órgãos genitais femininos (com 8,6%, cada), e de esôfago, colón e próstata (com 5,1%, cada).

Notaram-se também maior prevalência de câncer gástrico nessa amostra e predominância do sexo masculino (56,9%).

A mediana dos resultados obtidos do estresse oxidativo foi de 5,00μM, estando 63,8% dos indivíduos com valores elevados de estresse oxidativo. Quanto ao perfil lipídico dos pacientes, foram observados os seguintes fatores: a mediana dos triacilglicerois estava normal (138,78±89,88mg/dL), o colesterol total estava na classificação desejável (163,04±172,38mg/dL), o LDL-c estava no limiar elevado (151,30±178,25 mg/dL) e o HDL-c era baixo (31,70±22,74mg/dL) (Tabela 1).

**Tabela 1:** Distribuição dos pacientes com câncer acompanhados no Hospital Geral Dr. César Cals, de julho de 2011 a julho de 2012, de acordo com as variáveis estudadas

Variáveis	Mediana (n=58)	Grupo 1 n (%)	Grupo 2 n (%)	Valor de p*
Malondialdeído (μM)	5,00±4,07			
Normal	21 (36,2%)	9 (42,9)	12 (57,1)	0,412
Alto	37 (63,8%)	20 (54,1)	17 (45,9)	
Colesterol total (mg/dL)	163,04±172,32			
Desejável	42 (72,4%)	21 (50,0)	21 (50,0)	0,865
Limítrofe alto	5 (8,6%)	3 (60)	2 (40)	
Alto	11 (19,0%)	5 (45,5)	6 (54,5)	
HDL-c (mg/dL)	31,70±22,74			
Baixo	34 (58,6%)	22 (64,7)	12 (35,3)	0,019
Normal	14 (24,2%)	3 (21,4)	11 (78,6)	
Alto	10 (17,2%)	4 (40,0)	6 (60,0)	
Triacilglicerol (mg/dL)	138,78±89,88			
Normal	31 (53,5%)	17 (54,8)	14 (45,2)	0,688
Limítrofe	10 (17,2%)	4 (40,0)	6 (60,0)	
Alto	17 (29,3%)	8 (47,1)	9 (52,9)	
LDL-c (mg/dL)	151,30±178,25			
Ótimo	12 (20,7%)	6 (50,0)	6 (50,0)	0,296
Limiar ótimo	11 (19,0%)	3 (27,3)	8 (72,7)	
Limiar elevado a muito elevado	35 (60,3%)	20 (57,1)	15 (42,9)	

* Teste χ^2^. Grupo 1 formado por indivíduos com câncer gastrintestinal; Grupo 2 formado por indivíduos com outros cânceres. HDL-c: lipoproteína de alta densidade colesterol; LDL-c: lipoproteína de baixa densidade colesterol.

No entanto, 27,6% (16/58) dos pacientes com câncer apresentaram valores classificados como limítrofe alto ou alto de colesterol total; 58,6% (34/58) apresentaram valores baixos de HDL-c; e 29,3% (17/58) valores altos para os triacilglicerois (Tabela 1).

Na comparação entre o Grupo 1 (n=29), que apresentava câncer gastrintestinal e de órgãos anexos, com o Grupo 2 (n=29), de pacientes com os outros tipos de câncer, foi observada associação entre a maior prevalência de indivíduos do Grupo 1 com HDL-c baixo do que no Grupo 2 (p=0,019) (Tabela 1).

Em relação às diferenças dos valores medianos de HDL-c, o Grupo 1 apresentou níveis mais baixos (31,32mg/dL) do que pacientes oncológicos do Grupo 2 (43,67mg/dL) (p=0,038). Maiores valores de estresse oxidativo foram observados nos pacientes do Grupo 1, com 6,83µM, enquanto que o Grupo 2 apresentou 4,46µM (p=0,027) (Tabela 2).

**Tabela 2:** Valores medianos de malondialdeído e lipoproteínas plasmáticas dos pacientes com câncer acompanhados no Hospital Geral Dr. César Cals, de julho de 2011 a julho de 2012

Parâmetros bioquímicos	Grupo 1	Grupo 2	Valor de p*
Malondialdeído (µM)	6,83±4,92	4,46±2,57	0,027
Colesterol total (mg/dL)	209,36±161,73	210,82±185,30	0,975
Triacilglicerol (mg/dL)	141,85±86,36	146,48±94,76	0,878
HDL-c (mg/dL)	31,32±21,18	43,67±22,92	0,038
LDL-c (mg/dL)	206,40±171,24	196,45±187,90	0,834

* Teste *t *de Student. HDL-c: lipoproteína de alta densidade colesterol; LDL-c: lipoproteína de baixa densidade colesterol.

## DISCUSSÃO

O estudo realizado apresentou limitações importantes quanto à sua população e à sua amostra. A população definida ficou limitada somente a um local de estudo e não foi delimitado um tipo de neoplasia específica, o que poderia resultar em resultados com maior acurácia e aplicáveis a população em geral. Outra limitação importante é o tamanho da amostra, visto que não foi calculada uma amostra representativa, não podendo ser generalizada para indivíduos em termos gerais.

A maior prevalência de câncer gastrintestinal e a alta ocorrência de câncer nos órgãos genitais femininos na amostra ocorreram, provavelmente, por se tratar de um hospital terciário de alta complexidade de referência nas áreas de cirurgia, clínica médica, ginecologia, obstetrícia e neonatologia.^(17)^ O câncer gástrico acomete mais homens, chegando a uma proporção de 2:1, ocorrendo geralmente após os 40 anos de idade, possuindo um pico de incidência entre os 50 e 70 anos.

E sendo incidente nas classes com mais baixas condições socioeconômicas e de menor escolaridade.^(18)^

Em artigo de revisão sobre terapia nutricional no câncer gástrico, foi observado que existem dois grupos de fatores de risco para tal neoplasia: o constitucional, ou intrínseco e o ambiental, ou extrínseco. A dieta é colocada como fator de risco ambiental mais relevante,^(19)^ e o álcool também é mencionado como fator de risco, assim como o estado de hipocloridria ou acloridria por uso de medicamentos, que proporciona um pH estomacal mais propício à formação de colônias bacterianas, facilitando a infecção pelo *Helicobacter pylori*, que promove um processo inflamatório crônico, culminando no aparecimento da neoplasia. O estresse oxidativo induzido pela inflamação atua como mediador da formação ou ativação de partículas cancerígenas, provocando danos ao DNA e desempenhando um papel importante nesse processo da carcinogênese.^(20)^

Os resultados obtidos do estresse oxidativo apresentaram-se altos, com mediana de 5,00μM quando comparados ao valor de 3,31μM de indivíduos adultos saudáveis.^(14)^ Quando os mecanismos de controle de antioxidantes são esgotados ou saturados, o potencial de redox celular se desloca em direção ao estresse oxidativo, que, por sua vez, aumenta o potencial de dano para ácidos nucleicos, lipídios ou proteínas e, quando esses danos ao DNA não são reparados, podem resultar em mutação, estabelecendo o papel do dano oxidativo de DNA nuclear na neoplasia.^(21)^

Quanto ao perfil lipídico da população estudada, a mediana baixa de HDL-c condiz com a literatura. Em revisão sistemática, foi observada associação inversamente significativa entre HDL-c e a incidência do risco de câncer. Essa associação é independente do LDL-c, idade, índice de massa corporal (IMC), diabetes e tabagismo.^(22) ^Baixos níveis de HDL-c podem estar associados à epidemiologia do risco de câncer de pulmão e à progressão de tumores localizados para o metastático.^(23)^

O HDL-c é conhecido por seu efeito antiaterogênico no organismo e, juntamente da proteína C-reativa, apresenta características promissoras como preditor de mortalidade cardiovascular.^(24)^

O papel antiaterogênico exercido pelo HDL está atrelado ao transporte reverso de colesterol (RCT), incluindo ações antioxidantes, tanto *in vitro*, quanto *in vivo*, anti-inflamatórias e antitrombóticas.^(25,26) ^Tal atividade também está relacionada à presença de várias apolipoproteínas (Apo AI, apoE, apoJ, apoA-II e apo A-IV) e enzimas paraxonase sérica (PON1), FAP-acetil-hidrolase (FAP- AH), lecitina-colesterol aciltransferase (LCAT) e glutationa peroxidase (GSPx), com propriedades antioxidantes.^(27)^ No entanto, HDL pode ser modificada em certas patologias e essas modificações podem afetar suas funções.^(28)^

Ainda sobre a comparação entre os Grupos 1 e 2, foi observado um maior estresse oxidativo no Grupo 1, o que pode estar associado ao mau prognóstico dos cânceres de pâncreas e esôfago, relacionado à agressividade invasiva desses tumores. O câncer pancreático, apesar da baixa incidência, apresenta alta mortalidade, mesmo em diagnóstico precoce, assim como o câncer de esôfago, que possui uma sobrevida em 5 anos <10%.^(29)^

## CONCLUSÃO

A maioria da amostra era do sexo masculino e apresentou estresse oxidativo mais elevado do que indivíduos saudáveis, colesterol total desejável, LDL-colesterol no limiar elevado e HDL-colesterol baixo. Essa última lipoproteína foi classificada também como mais baixa ainda entre os pacientes com câncer gastrintestinal e órgãos anexos. Esses pacientes apresentaram, também, maiores níveis de estresse oxidativo. Portanto, o perfil lipídico apresentou-se desfavorável, principalmente em relação ao LDL-colesterol e ao HDL-colesterol, o que parece ter contribuído para uma maior taxa de peroxidação lipídica. Novos estudos são necessários para esclarecer as possíveis relações entre estresse oxidativo e perfil lipídico no câncer gastrintestinal.
